# A novel tumour suppressor lncRNA F630028O10Rik inhibits lung cancer angiogenesis by regulating miR‐223‐3p

**DOI:** 10.1111/jcmm.15044

**Published:** 2020-02-13

**Authors:** Limei Qin, Menglong Zhong, Dickson Adah, Li Qin, Xiaoping Chen, Chunquan Ma, Qiang Fu, Xiaoping Zhu, Zhili Li, Nina Wang, Yanfeng Chen

**Affiliations:** ^1^ Guangdong Provincial Key Laboratory of Animal Molecular Design and Precise Breeding Foshan University Guangdong China; ^2^ School of Life Science and Engineering Foshan University Guangdong China; ^3^ State Key Laboratory of Respiratory Disease Guangzhou Institutes of Biomedicine and Health Chinese Academy of Science Guangdong China

**Keywords:** angiogenesis, F630028O10Rik, LncRNA, lung cancer, miR‐223‐3p

## Abstract

Lung cancer is the world's leading cause of cancer‐related morbidity and mortality despite advances in surgery, chemotherapy and immunotherapy; thus, there is an urgent need to find new molecules to develop novel treatment strategies. Although ncRNAs were found to account for 98% transcripts, the number of lncRNAs with distinct function in lung cancer is extremely limited. We previously demonstrated that Plasmodium infection inhibits tumour growth and metastasis, but the exact mechanisms involved have not been fully understood. In this study, we carried out RNA sequencing (RNA‐Seq) of tumour tissues isolated from LLC tumour‐bearing mice treated with either Plasmodium yoelli (Py)‐infected red blood cells or uninfected red blood cells. We found that F630028O10Rik (abbreviated as F63) is a novel lncRNA that was significantly up‐regulated in tumours isolated from mice treated with Py‐infected red blood cells compared to the control. By using gene silencing technique, F63 was found to inhibit both tumour Vascular Endothelial Growth Factor A (VEGFA) secretion and endothelial cells clone formation, migration, invasion and tube formation. Injection of cholesterol‐modified siRNA‐F63 into mice tumour tissues produced a significant increase in tumour volume, blood vessel formation and angiogenesis 17 days after injection. We further showed that inhibiting miR‐223‐3p results in the down‐regulation of VEGFA and VEGFR2 which are vital molecules for angiogenesis. These results reveal that F63 inhibit tumour growth and progression by modulating tumour angiogenesis suggesting F63 can be a novel lncRNA with great potential as a candidate molecule for gene therapy in lung cancer.

## 1 INTRODUCTION

Cancer is the major cause of death globally and is estimated to account for 9.6 million deaths in 2018, among which lung cancer is the most common,^1^ and Non–small‐cell lung cancer (NSCLC) accounted for about 85%. Like many other cancers, many NSCLC patients are diagnosed at an advanced stage and 5‐year survival rate is only 15.9%.^2^ Therefore, lung cancer is the malignant tumour with the highest morbidity and mortality in the world. With the development of medical technology, molecular targeted therapy and immunotherapy have become new means of treatment for NSCLC, but the 5‐year survival rate of NSCLC patients was only slightly improved. Hence, adequate understanding of crucial molecular processes during lung cancer development is the key to developing novel gene therapy strategy against lung cancer.

Numerous studies have shown that the expression level of some lncRNAs in metastatic tissues was significantly different from that in primary and normal tissues of cancer patients. LncRNA therefore has been given more particular attention in the cancer research. LncRNAs that can inhibit tumour development are called ‘tumour‐suppressing lncRNAs’. Discovering new tumour‐suppressing lncRNAs and elucidating their functions is important for understanding the mechanism of tumorigenesis and is the premise to further research and development of new therapeutic targets. Meg3 is the first lncRNA that was identified as having tumour suppressive function by inhibiting the proliferation of cancer cells and modulating the Rb pathway. Some anti‐lung cancer medicines like Pabosini can activate the Rb pathway and increase the expression of Meg3 in A549 and SK‐MES‐1 lung cancer cells, which provides a potential method for the treatment of lung cancer.^3^ Therefore, tumour‐suppressing lncRNAs have potential clinical application in the treatment of NSCLC.

In the current study, a lncRNA named F630028O10Rik (abbreviated as F63) was found to be differentially expressed in our animal model of lung cancer. Previous studies have reported that nonlethal plasmodium parasite (i.e. Py infection) could suppress lung cancer^4^, ^5^ as well as other tumours such as liver cancer.^6^ In order to establish the suppressive model of lung cancer, mice were injected with LLC cells (s.c.) as well as Py (i.p), while mice injected with LLC cells (s.c.) alone served as the control group. Seventeen days post‐Py infection, evaluation of lncRNAs in tumour tissues was performed by the next‐generation sequencing. Interestingly, a lncRNA named F630028O10Rik was found to be significantly up‐regulated in the Py‐treated tumour‐bearing mice compared with LLC (control) group. By using subcellular location and pull down assay, we found that F63 mainly locates in the nucleus and interacts with many proteins that are associated with angiogenesis and blood vessel development; we thus hypothesized that F63 could modulate angiogenesis during tumour development.

## 2 MATERIALS AND METHODS

### 2.1 Cells, parasite and animals

The lung cancer cell line, LLC, and mouse endothelium cell line, MS1, were obtained from the American Type Culture Collection (ATCC), cultured in DMEM (HyClone) supplemented with 10% foetal bovine serum (HyClone). C57BL/6 (6‐week‐old) mice were obtained from the Guangdong medical laboratory animal centre. The nonlethal Plasmodium yoelii 17XNL (Py) strain was obtained from the Malaria Research and Reference Reagent Resource centre (MR4).

### 2.2 Animal model for RNA‐Seq

C57BL/6 mice were subcutaneously (s.c.) injected with 5 × 10^5^ LLC cells and were randomly divided into two groups (n = 8 per group). LLC tumour‐bearing mice injected with 5 × 10^5^ Py‐infected red blood cells (Py‐RBCs) served as the Py + LLC group, while LLC‐bearing mice injected with uninfected RBCs served as LLC (control) group. Seventeen days post‐injection with Py‐RBCs, three mice from each group were selected for tumour tissue isolation and total RNA isolation, and were then sent to Biotechnology Corporation for Long non‐coding RNA sequencing.

### 2.3 Subcellular localization

The cytoplasm and nucleus of LLC cells were separated by Nuclear/Cytosol Fractionation Kit (Biovision) and followed with RNA extraction, reverse transcription and real‐time fluorescent quantitative PCR.

### 2.4 RNA pulldown and liquid chromatography‐mass spectrometry (LC‐MS)

Biotin‐labelled F63 was transcribed in vitro with the Biotin RNA Labeling Mix (Roche) and T7 RNA polymerase (Roche) and then treated with RNase‐free DNase I (Roche) and 0.2 mol/L EDTA to stop the reaction. Biotinylated RNAs were mixed with streptavidin agarose beads (Life Technologies) at 4℃ overnight. Total cell lysates and RNase inhibitor were added to each binding reaction and incubated on ice for 1 hour. The RNA‐protein binding mixture was boiled in SDS buffer, and the eluted proteins were analysed by LC‐MS, which was performed by Luming Biotechnology.

### 2.5 Bioinformatics analysis

After mass spectrometry, the genes corresponding to the identified proteins were analysed by KEGG signalling pathway using online bioinformatics software David (https://david.ncifcrf.gov/tools.jsp). The binding sequences of F63 and miR‐223 were predicted by online bioinformatics software Hybrid (https://bibiserv.cebitec.uni-bielefeld.de/rnahybrid).

### 2.6 qRT‐PCR analysis

Total RNA was extracted using the TRIzol reagent (Thermo Fisher). RNA was reverse‐transcribed into cDNA with a Reverse Transcription Kit (Takara). Real‐time PCR was performed using the SYBR Premix Ex Taq (TaKaRa) following the manufacturer's instructions. The results were normalized to β‐actin. The fold change was calculated using 2^−ΔΔCT^. The primers used for qRT‐PCR were as follows:

F63 forward: 5′ CTTTGTTGCTGTTGCTCTCCC 3′,
F63 reverse: 5′ TGTCCACCCCACCACTTTTTC 3′.
β‐actin forward: 5′ AACAGTCCGCCTAGAAGCAC 3′
β‐actin reverse: 5′ CGTTGACATCCGTAAAGACC 3′
VEGFA forward: 5′ GACATCTTCCAGGAGTACC 3′
VEGFA reverse: 5′ TGCTGTAGGAAGCTCATCT 3′,
VEGFR2 forward: 5′ CAGTGGGATGGTCCTTGCAT 3′
VEGFR2 reverse: 5′ ACTGGGCATCATTCCACCAA 3′

### 2.7 Western blot assay

VEGFA and β‐actin and Tubulin antibodies were purchased from Novus Biologicals and Cell Signaling, respectively. The protein lysates for all cells were separated using 12% SDS PAGE. Later, they were transferred onto 0.22‐μm PVDF membranes (Millipore) and then incubated with VEGFA antibody. Anti β‐actin served as control. Proteins were detected with Super Signal Chemiluminescence Substrate (Pierce, Thermo Scientific).

### 2.8 Transient transfection and RNA interference in cells

F63‐specific siRNA (siRNA‐F63) and siRNA‐control (siRNA‐NC), miR‐223‐3p mimic and inhibitor were purchased from Ribobio. The sequences of the siRNA‐F63 were Forward: 5′ GUGUCUAGCUCUCCUUACATT 3′, Reverse: 5′ UGUAAGGAGAGCUAGACACTT 3′, siRNA‐NC was offered by Riobio company. All the siRNA were transfected at 50 nmol/L into LLC and MS1 cells using Lipofectamine 3000 (Invitrogen) according with the manufacturer's guidelines. Both of the mimic of miR‐223‐3p and mimic‐NC were transfected into the LLC at 50 nmol/L; Both of the inhibitor of miR‐223‐3p and inhibitor‐NC were transfected into the LLC at 100 nmol/L. Total RNA was extracted 24 hours after transfection for qRT‐PCR, total protein was extracted 48 hours after transfection for Western blot.

### 2.9 siRNA injection into tumour of mice

Methoxy‐2‐tetralone and cholesterol‐modified F63‐specific siRNA (siRNA‐F63), siRNA‐control (siRNA‐NC) were purchased from Ribobio. The sequences of the siRNA‐F63 were described above. The siRNA‐F63 and siRNA‐NC were intratumour‐injected into the tumour‐bearing mice every other day. The tumour sizes were measured every two days; mice were killed at 17 day for tumour tissues isolation.

### 2.10 Colony formation assay

MS1 cells (n = 800) which have been transfected with siRNA‐F63 for 24 hours were placed into six‐well plates and maintained in media for 2 weeks. The medium was replaced every 4 days. Colonies were fixed with methanol and stained with 0.1% crystal violet (Sigma‐Aldrich) for 15 minutes. The visible colonies were then counted. All experiments were performed three times in triplicate.

### 2.11 Cell migration and invasion assays

For the migration assays, at 24 hours post‐transfection, 5 × 10^4^ cells in serum‐free media were placed into the upper chamber of an insert (8‐μm poresize; Millipore). For the invasion assays, at 24 hours post‐transfection, 1 × 10^5^ cells in serum‐free medium were placed into the upper chamber of an insert coated with Matrigel (BD Biosciences). Medium containing 10% foetal bovine serum was added to the lower chamber. After incubation for 24 hours, the cells remaining on the upper membrane were removed with cotton wool. Cells that had migrated or invaded through the membrane were stained with methanol and 0.1% crystal violet. The stained cells were counted under microscope (Olympus Corp.) in six randomly selected fields and the number of cells was presented relative to untreated controls. All experiments were conducted three times in triplicate.

### 2.12 Wound healing assay

SiRNA treated cells were placed in a 24‐well plate at the density of 1 × 10^6^ for six wells in each groups. A wound line was scratched vertically to the bottom with a 200‐μL pipette tip after the cells were grown to 100% confluence. Cells were washed twice with PBS and cultured for 24 hours, three randomly selected fields were photographed at the beginning and end of treatment. Then cells were washed with PBS and fixed with 4% formaldehyde and then stained with crystal violet for 30 minutes. Phase images were taken under the microscope at 100 magnification. The average width of each wound was calculated by micro imaging software cell Sens Standard (Olympus).

### 2.13 Endothelial cell tube formation assay

Plates were coated using Matrigel (Cat. No. 354234, Corning) according to the manufacturer's instructions. Fifteen microlitres of matrigel was applied to each well of 96‐well plates and incubated at 37℃ for 30 minutes. 1 × 10^4^ different siRNA treated MS1 cells were plated in each matrigel substratum containing 10% FBS. Cells were then incubated for 6 hours at 37℃ in a 5% CO_2_ humidified atmosphere. Images were taken using Olympus microscope at 200 magnification, and the number of tube branching points per field was quantified using ImageJ software. Three fields were randomly selected in each well.

### 2.14 In vivo tumorigenicity

A 5‐week‐old male C57BL/6J mice were randomly divided into three groups, with four mice in each group. LLC cells (5.0 × 10^5^) were injected subcutaneously into the right flanks of the mice. The siRNA‐F63 (200 nmol/L), siRNA‐NC (200 nmol/L) and saline (equal amount) were injected into the tumour every 2 days, respectively. The developing tumours were observed. After 17 days, the primary tumours were excised, paraffin‐embedded and formalin‐fixed, followed by Haematoxylin and Eosin (H&E) staining and immunostaining to analyse the expression of CD31, in accordance with the manufacturer's instructions.

### 2.15 Immunohistochemistry analysis

Fresh tumours were fixed with 10% neutral formalin for 3 day and then embedded in paraffin. The tissues were sectioned at the thickness of 4 μm. Immunohistochemistry staining for CD31 was performed using the Histostain‐Plus Kit (Kangwei). After sequential treatments, tissue sections were incubated with normal serum for 30 minutes, then incubated with the control IgG and primary antibodies against CD31 (Abcam, 1:200) at 4℃ overnight and then HRP‐labelled secondary antibody for 30 minutes. The 3,3‐diaminobenzidine (DAB) solution was used for development of brown colour to identify the expression of CD31.

### 2.16 Dual‐luciferase reporter assay

The predicted binding sequences between F63 and mmu‐miR‐223‐3p were amplified by PCR and subsequently inserted into the polyclonal site of vector psiCHECK™‐2, a dual fluorescence reporting vector, to obtain the recombinant vector. The recombinant vector and mmu‐miR‐223‐3p mimics or mimic‐control (RiboBio) were cotransfected into 293T cells, respectively. After 6 hours, the cells were lysed (Dual‐Glo^®^ Luciferase Assay System, Lot^#^E2920, Promega) for chemiluminescence on Multiscan Spectrum.

### 2.17 Statistical analysis

The experimental results were presented as mean ± SD. The statistical differences among different groups were analysed by the ANOVA test for multiple comparisons and student's *t* test respectively. *P* value < .05 was considered statistically significant. Statistical analyses were performed with the SPSS 19.0 software (SPSS).

## 3 RESULTS

### 3.1 F63 was dysregulated in lung cancer mice model

**Figure 1:**
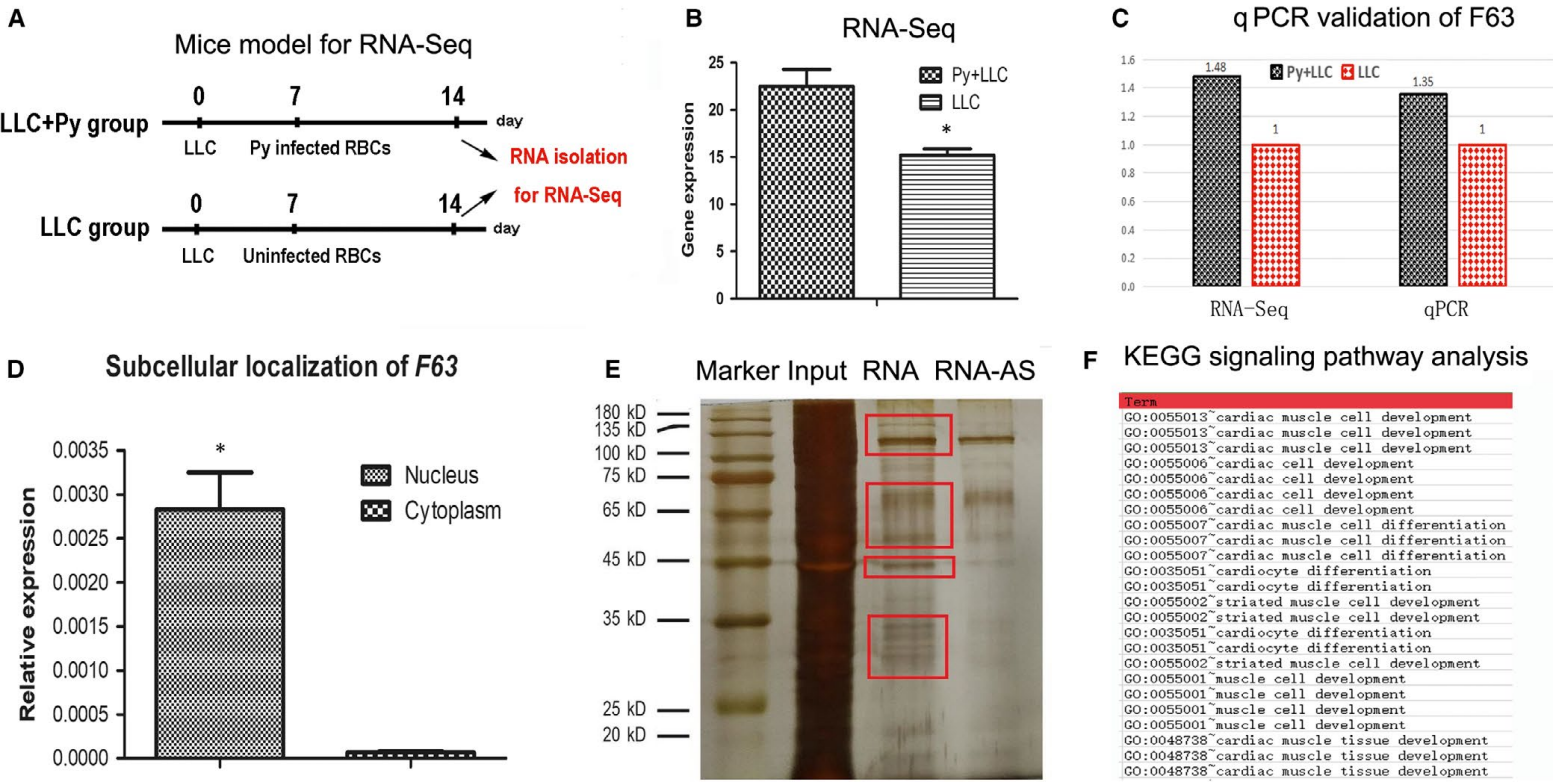
Plasmodium‐mediated dysregulation of F63 in mice lung cancer. A, Design of animal experiment for RNA sequencing. B, RNA‐Seq data of Py + LLC group (Py‐treated group) and LLC group (control group). C, qPCR validation of RNA‐Seq data of lncRNA F63. D, Subcellular localization of lncRNA F63. E, SDS page of RNA‐protein binding mixture. Input: RNA‐protein binding mixture; RNA: F63 probe; RNA‐AS: anti‐sense of F63 probe as a control; red boxes indicated the regions that were different compared with control. F, KEGG pathway enrichment of proteins identified in the mass spectrometry of the red boxes. Asterisk: *P* < .05

In our previous work, we constructed lung cancer mice model using plasmodium parasite, the mice were divided into two groups, one group was injected with LLC cell as well as plasmodium parasite (Py + LLC), while the other group was injected with lewis lung cancer (LLC) cells to serve as control. Seventeen days later, the tumour volume was found to be significantly smaller than the control group (Figure 1A). Subsequently, the tumour samples were sent to next‐generation sequence for lncRNA profiling, among the 5960 differential expressed lncRNAs, 5562 lncRNAs were up‐regulated in Py + LLC group compared with LLC group; 398 lncRNAs were down‐regulated in Py + LLC group compared with LLC group. Several most differentially expressed lncRNAs were selected for elementary experiments via siRNA technology, we found one lncRNA—F63 deserved for further study for the following reasons. Firstly, F63 was significantly up‐regulated in the Py‐treated group compared with control group (Figure 1B), which was subsequently confirmed by quantitative polymerase chain reaction (qPCR) (Figure 1C). To better understand this lncRNA, secondly, we isolated the nucleus and cytoplasm and tested the expression of F63 and found that F63 was abundantly expressed in the nucleus (Figure 1D). Subsequently, in order to learn more about F63, we carried out pull down assay to investigate what kind of proteins could interact with F63. Using specific probe, we found four bands were different compared with control groups (Figure 1E), we then selected these bands for mass spectrum identification. The identified proteins were analysed by bioinformatics and enriched by KEGG signalling pathway, which reveals its involvement in cardiac cell development, cardiocyte differentiation, cardiovascular system development, heart development, etc (Figure 1F).

### 3.2 F63 inhibition promotes VEGFA at both transcription and translation level

**Figure 2:**
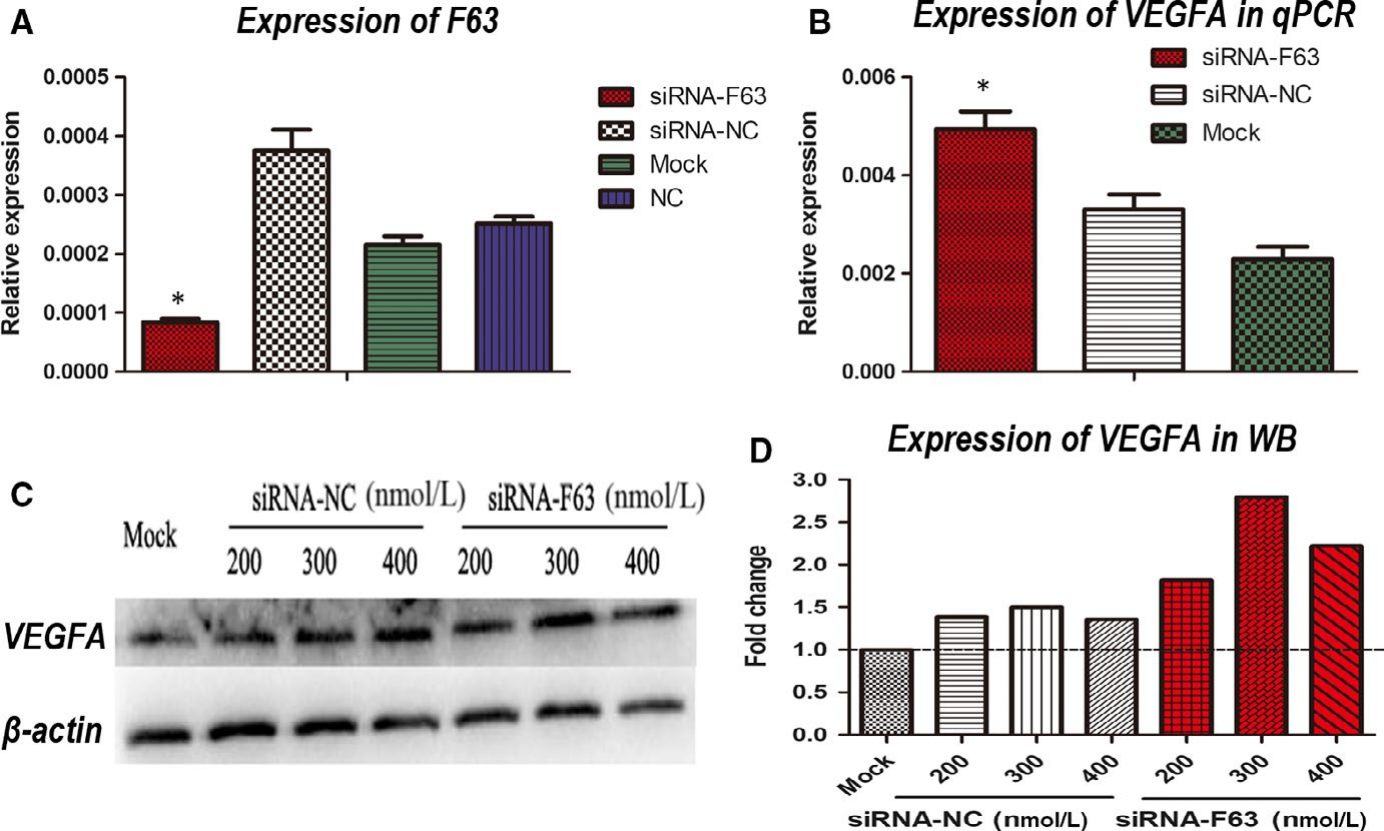
F63 inhibition promotes VEGFA at both transcription and translation level. A, qPCR analysis of F63 expression after siRNA‐F63 and siRNA‐NC transfection into LLC. B, qPCR analysis of VEGFA expression after siRNA‐F63 and siRNA‐NC transfection into LLC. C, Western blot analysis of VEGFA expression with β‐actin as housekeeping gene. D, Fold change of VEGFA expression in comparison with β‐actin after siRNA‐F63 and siRNA‐NC transfection into LLC using different concentrations. Asterisk: *P* < .05

To validate if F63 could modulate tumour angiogenesis, we used a cholesterol‐modified siRNA‐F63 to transfect lewis lung cancer cells (LLC) while siRNA‐NC served as control. Compared with siRNA‐NC group, a remarkable down‐regulation of F63 at transcription level was observed (Figure 2A). Using qPCR, the transcription level of VEGFA was tested 24 hours later, and we found a significant up‐regulation of VEGFA in the siRNA‐F63 group compared to the control groups (Figure 2B). We further tested VEGFA at translation level using Western blotting, and the same trend of up‐regulation of VEGFA was observed (Figure 2C,D), indicating that F63 could suppress the expression of VEGFA at both transcription and translation level.

### 3.3 F63 inhibition impacts vascular endothelial cells

**Figure 3:**
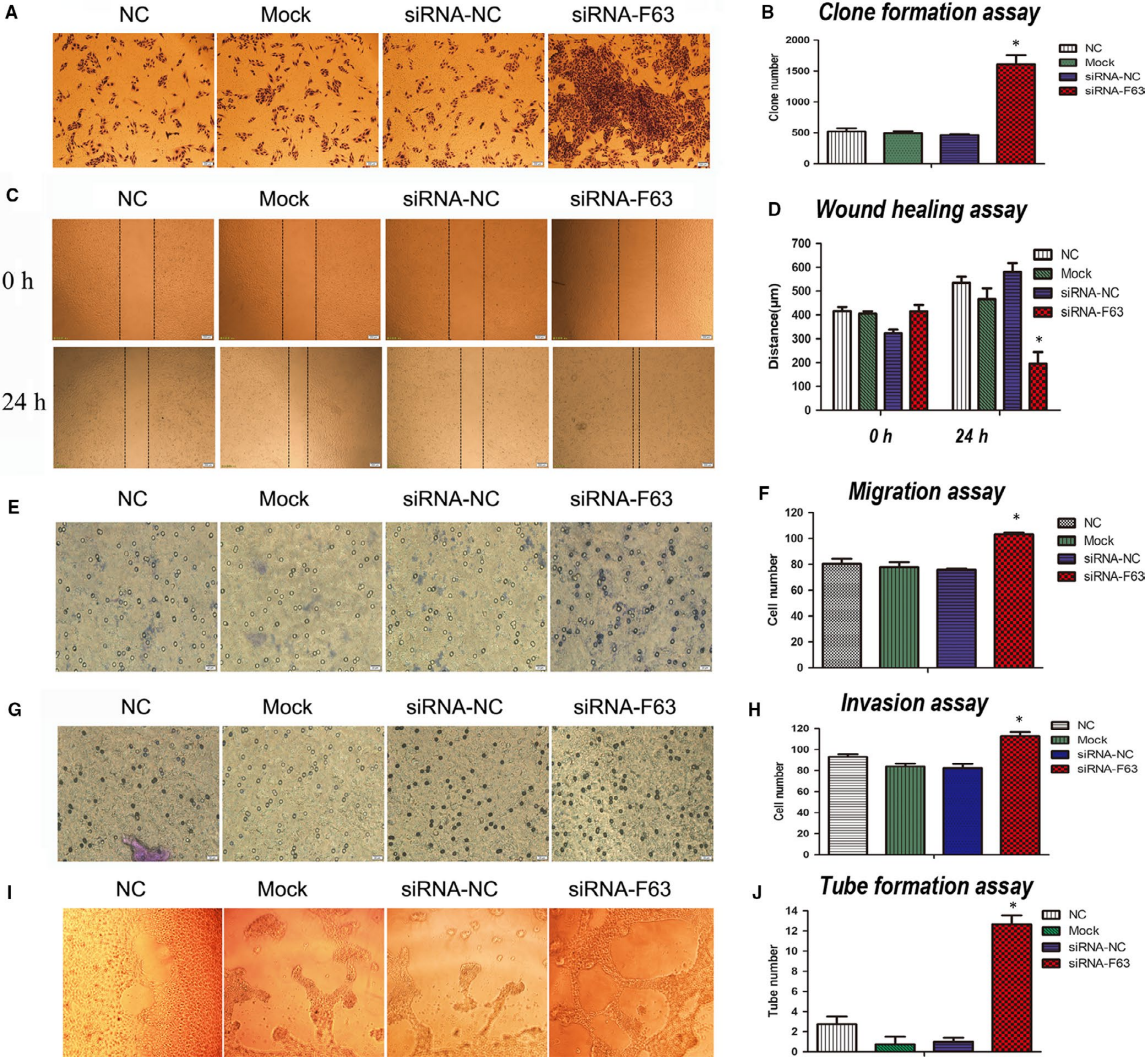
Impact of F63 inhibition on vascular endothelial cell. Representative results of the impact of F63 inhibition on A, Clone formation; C, Wound healing; E, Migration; G, Invasion and I, Tube formation and their respective quantification (B, D, F, H, J) in MS1 cells. Asterisk: *P* < .05.Clone formation assay (40 × magnification), wound healing assay (40 × magnification), migration/invasion assay (400 × magnification), tube formation assay (200 × magnification)

To test the impact of F63 on vascular endothelial cell, we used MS1 to perform the following experiments. siRNA‐F63 was transfected into MS1 cells, siRNA‐NC and liposome alone (Mock) and NC (cell alone) were set as controls. In the clone formation assay, siRNA‐F63 significantly increased the clone number of MS1 compared with the control groups (Figure 3A,B). In the wound healing assay, siRNA‐F63 significantly decreased the distance of the wound compared with the control groups (Figure 3C,D). In the migration assay, siRNA‐F63 significantly increase the number of MS1 cells which migrated through the membrane compared with the control groups (Figure 3E,F). In the invasion assay, siRNA‐F63 significantly increased the number of MS1 which have migrated through the matrigel and membrane compared with the control groups (Figure 3G,H). In the tube formation assay, siRNA‐F63 significantly increased the number of tubes formed compared with the control groups (Figure 3I,J). Taken together, the above experiments demonstrated that F63 could inhibit clone formation, migration, invasion and tube formation in vascular endothelial cells, which could further influence angiogenesis in tumours.

### 3.4 Inhibition of F63 could promote tumour angiogenesis in vivo

**Figure 4:**
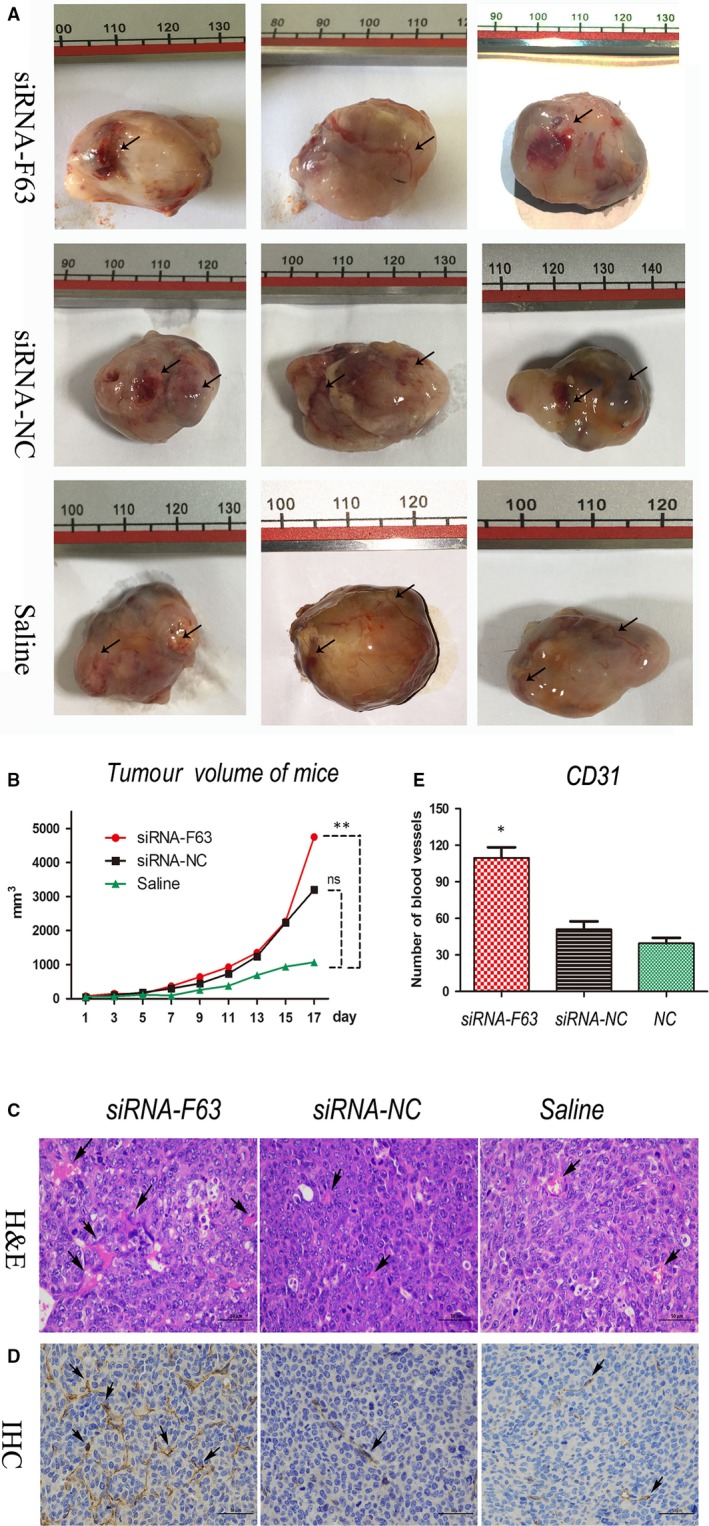
F63 inhibition promotes tumour angiogenesis in vivo. A, Tumour tissues in LLC lung tumour‐bearing mice after injection of siRNA‐F63 and control treatment. Arrows show the blood vessels in the tumour tissues; unit of vernier caliper is millimetre. B, Mice tumour volumes at day 17 after injection of siRNA‐F63 and control treatment. C, H&E staining (400 × magnification) and D, CD31 immunohistochemical assay (IHC, magnification) and E, Quantification of IHC in tumour tissue sections. Asterisk represented the *P* value was less than .05 compared with other groups. Asterisk: *P* < .05; Double asterisks: *P* < .01

As a hypothesis, F63 play a negative role in angiogenesis of tumour, thus inhibition of F63 by siRNA might promote the growth of blood vessels in tumour. To validate this hypothesis, we carried out the in vivo study on mice. Our animal experiment showed that the blood vessels in tumours of siRNA‐F63 group were much thicker in diameter and particularly bright red in colour; In contrast, the blood vessels in tumour of siRNA‐NC as well as Saline group were quite smaller in diameter and dim in colour (Figure 4A). These results showed that F63‐inhibition promoted the blood vessels development in mice. Furthermore, the tumour volume of siRNA‐F63 groups was significantly larger than the control groups at day 17 (Figure 4B), which demonstrated that F63‐inhibition could gradually promote tumour volume in the later stage of tumour development.

To further assess the effect of siRNA‐F63 on the development of new blood vessels in tumour tissues, we carried out a tissue section and H&E staining. It was obvious that more blood vessels were found in siRNA‐F63 group compared with the control groups (Figure 4C, see arrows). CD31 is a platelet endothelial cell adhesion molecule, which is commonly used for evaluation of tumour angiogenesis. By using immunohistochemistry (IHC) assay, we observed a significant increase in blood vessel formation in the siRNA‐F63 group compared to the controls (Figure 4D, see arrows). Analysis of the immunohistochemistry results indicates a significant difference in CD31 expression between the groups (Figure 4E). These results showed that F63‐inhibition led to the up‐regulation of VEGFA and promoted tumour blood vessel, formation with a resultant tumour enlargement ultimately suggesting that F63 can suppress tumour angiogenesis.

### 3.5 F63 interacts with miR‐223‐3p and leads to VEGFA and VEGFR2 suppression

**Figure 5:**
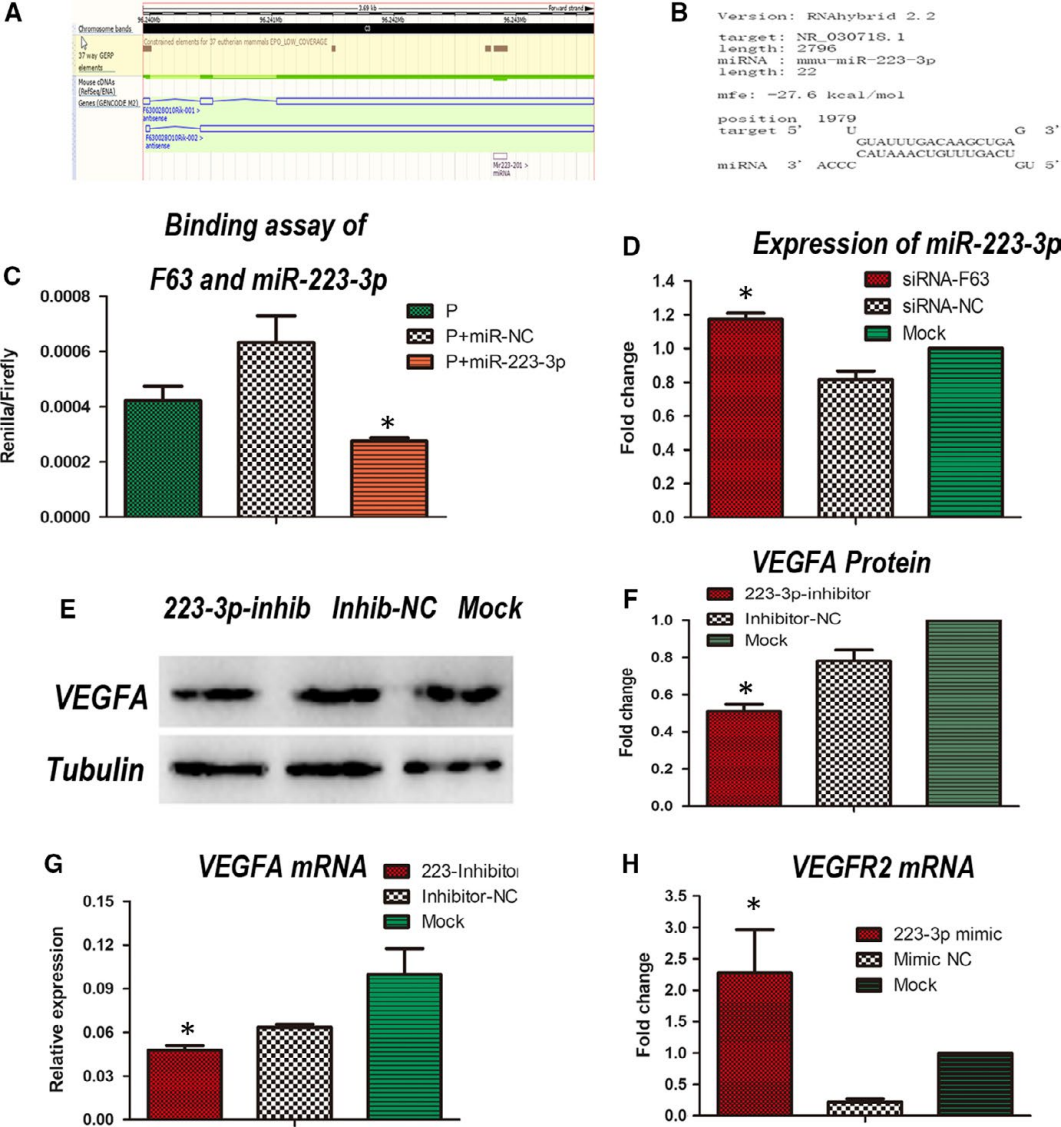
F63 interacts with miR‐223‐3p and leads to VEGFA suppression. A, Genome sequence demonstrating the overlap of miR‐223‐3p and F63. B, The binding site and free energy between F63 and miR‐223‐3p predicted by RNA hybid software. C, The dual‐luciferase reporter assay between F63 and miR‐223‐3p. P: Plasmid of dual‐luciferase reporter vector. D, qPCR of miR‐223‐3p after siRNA‐F63 and control treatments in LLC. E, Western blot of VEGFA and Tubulin and their F, Quantitative analysis. G, qPCR of VEGFA after miR‐223‐3p inhibitor and control treatments in LLC. H, qPCR of VEGFR2 after miR‐223‐3p mimics and control treatment in LLC. Asterisk represented the *P* value was less than .05 compared with other groups

**Figure 6:**
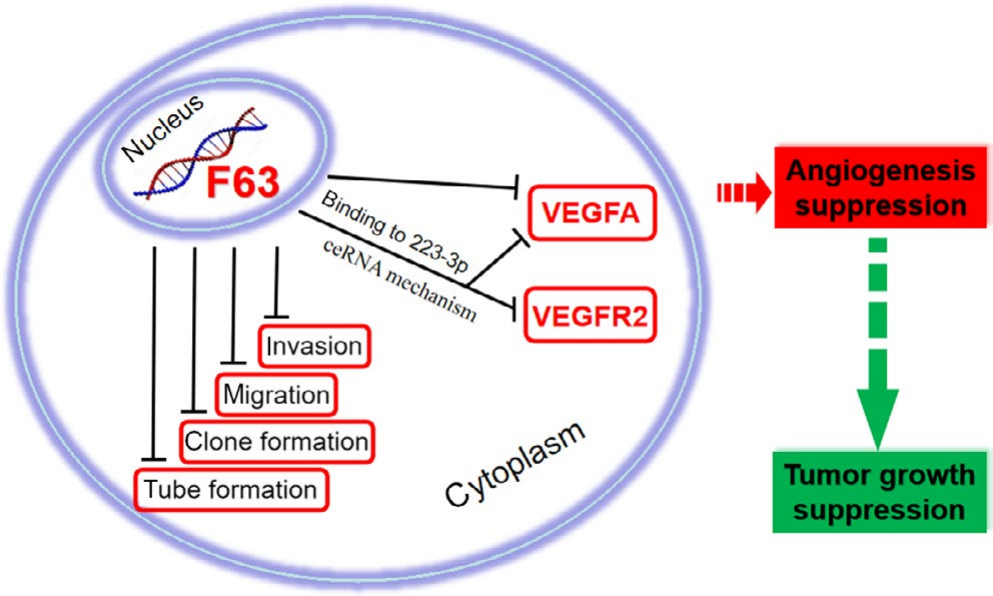
Schematic diagram of the interaction between F63, miR‐223‐3p and angiogenesis

As the genomic DNA of F63 and miR‐223‐3p overlaps (Figure 5A), we predicted the existence of a binding site using the online software, RNA‐hybrid 2.2. Result showed that F63 could bind to miR‐223‐3p at the position 1979 bp, the mfe was −27.6 kcal/mol (Figure 5B). To validate if F63 could bind to miR‐223‐3p directly, the dual‐luciferase reporter assay was carried out. The binding site was inserted into the luciferase vector, this recombinational plasmid was cotransferred into HEK 293T cells with the mimic of miR‐223‐3p. Results showed a significant decrease in luciferase activity, indicating that miR‐223‐3p actually binds to F63 via the predicted binding site (Figure 5C). To investigate whether or not F63 could influence the expression of miR‐223‐3p, we silenced F63 using siRNA‐F63, interestingly, the expression of miR‐223‐3p increased significantly (Figure 5D), suggesting that F63 could suppress the expression of miR‐223‐3p. To test if miR‐223‐3p could influence the angiogenesis signalling, miR‐223‐3p inhibitor and mimic was utilized. Result showed that inhibition of miR‐223‐3p could decrease the expression of VEGFA significantly at transcription level (Figure 5G) as well as translation level (Figure 5E,F), overexpression of miR‐223‐3p could increase the expression of VEGFR2 significantly (Figure 5H). These results indicated that F63/miR‐223/VEGF axis does play an important role in F63 inhibition of tumour vessels (Figure 6).

## 4 DISCUSSION

Over the past twenty years, long non‐coding RNAs (lncRNAs) have been demonstrated to be involved in diverse cellular processes, including regulation of gene expression, nuclear organization and nuclear‐cytoplasm trafficking through complex mechanisms.^7^ As many studies have shown, the specific function of lncRNA was closely related to the subcellular localization of lncRNA. Some lncRNAs are located in cytoplasm, for example lncRNA Ptenp1 is located in the cytoplasm and regulate the tumour suppressor, Pten^8^, ^9^; LncRNA Tincr is also located in cytoplasm and interacts with mRNA to increase their stability^10^; In addition, other lncRNAs such as Ror,^11^ HULC,^12^ linc‐MD1,^13^ 1/2‐sbs RNAs,^14^ and Gadd7^15^ are also located in the cytoplasm and play important functions in biological processes. On the other hand, some lncRNAs are located in the nucleus, for example lncRNA Heih is located in the nucleus and promotes tumour progression by recruiting PRC2^16^; LncRNA Let is located in the nucleus, and repression of Let by histone deacetylase 3 is shown to contribute to hypoxia‐mediated metastasis^17^; Besides, other lncRNAs such as Hotair,^18^ Malat1,^19^ Evf‐2,^20^ Lethe^21^ and Xist^22^ are located in the nucleus and take part in various processes including physiological and pathological processes, further suggesting that the specific function of lncRNAs are closely related to their subcellular localization.^23^ To clarify the initial functions of F63, subcellular localization of F63 in Lewis lung cancer cell was elucidated. It appeared that F63 was predominantly localized in the nucleus, which indicated that this lncRNA might regulate other gene expression by interacting with them directly or indirectly. Subsequently, we carried out pull down assay and found that F63 can directly bind to many angiogenesis‐related proteins, and this encouraged us to further study the functions and mechanisms of F63. We therefore hypothesized that F63 may inhibit tumour progression by affecting angiogenesis.

With the application of RNA sequencing as well as bioinformatics technologies, many abnormal expression of lncRNAs have been found by comparing the tumours of NSCLC patients with normal adjacent tissues. The abnormally up‐regulated expression of lncRNAs in NSCLC is usually classified as carcinogenic lncRNA. There are many functions of carcinogenic lncRNAs including: (a) maintaining cell growth and proliferation; (b) avoiding growth inhibitors; (c) ensuring continuous replication of DNA; (d) promoting metastasis and invasion; (e) inhibiting cell apoptosis and (f) inducing angiogenesis.^24^ For example, lncRNA Hotair can negatively regulate chromosomal transcription, recombine chromatin and promote cancer progression. Inhibition of Hotair could result in tumour suppression.^25^ On the other hand, the abnormally down‐regulated expression of lncRNAs in normal adjacent tissues is usually classified as anticancer lncRNA, they perform functions contrary to carcinogenic lncRNAs mentioned above. For example, lncRNA IGF2AS was found to be down‐regulated in NSCLC tumour tissue and was closely related to the overall survival of patients; IGF2AS therefore is an anticancer lncRNA and an ideal target for drug development.^26^ In the current study, F63 seems to belong to cancer suppressor lncRNA, as we found the most important gene for angiogenesis—VEGFA was up‐regulated significantly after inhibiting F63 by siRNA not only at transcription level but also at translation level. This phenomenon also appeared in lung tumour‐bearing mice model because the colour of tumour tissues was much more flammulated in F63‐siRNA group than that of the control groups, which presented the blood vessels in the tumour of siRNA‐F63 group are much more in number. Besides, the expression of CD31, which is a classical biomarker of angiogenesis, was up‐regulated in immunohistochemistry assay. Most interesting, inhibition of F63 could accelerate the growth of tumours in the late stage of tumour growth. Taken together, these results suggested that F63 is closely associated to angiogenesis, which leads to affect tumour growth in the end. To further explore the effect of F63 on angiogenesis, we carried out a series of experiments in mouse endothelial cells, the results confirmed that F63 could inhibit the proliferation, invasion, migration, tube formation of endothelial cells, a kind of cell that is essential for angiogenesis. All in all, for the first time, we confirmed our previous hypothesis, that is F63 actually inhibits angiogenesis and gradually plays a role in inhibiting lung cancer tumorigenesis.

Small non‐coding RNAs, that is microRNAs (miRs) are known to regulate the expression of target genes at the post‐transcriptional levels in tumour growth and propagation. A large amount of studies revealed that lncRNA exert a competing endogenous RNA (ceRNA) mechanism by inhibition miRNAs. For instance, lncRNA NR2F2‐AS1 influences the NSCLC cell proliferation, invasion and apoptosis through regulating miR‐320b targeting BMI1.^27^ LncRNA KCNQ1OT1 enhances the methotrexate resistance of colorectal cancer cells by regulating miR‐760.^28^ LncRNA Meg3 inhibits the progression of prostate cancer by modulating miR‐9‐5p.^29^ LncRNA SNHG20 promotes the tumorigenesis of oral squamous cell carcinoma via targeting miR‐197.^30^ In current study, lncRNA F63 may interact with miR‐223‐3p by directly binding in sequence, it seems the expression mode of F63 and miR‐223‐3p is adverse, as inhibiting F63 by siRNA cause an increase expression of miR‐223‐3p, interestingly, miR‐223‐3p seems to up‐regulate the expression of VEGFA and VEGFR2, because inhibition of miR‐223‐3p could suppress the expression of VEGFA, while overexpression of miR‐223‐3p could promote the expression of VEGFR2. These evidence suggested that F63 could bind to and suppress miR‐223‐3p, which in turn suppress the expression of VEGFA and VEGFR2, the vital moleculars during angiogenesis. As been reported by literatures, miR‐223‐3p was found to have bidirectional function in tumour progression, some studies found that miR‐223‐3p inhibited cancer cell proliferation, migration or invasion in nasopharyngeal carcinoma cells or hepato‐carcinoma cells.^31^, ^32^ Other studies found the opposite effect, that is miR‐223‐3p promotes cell proliferation, migration or invasion in helicobacter pylori‐associated gastric cancer cell or colorectal cancer, glioblastoma cells and renal carcinoma cell.^33^, ^34^, ^35^, ^36^, ^37^ As for angiogenesis in tumour progress, some studies found miR‐223‐3p could inhibit angiogenesis after detecting VEGFR2 and CD31 staining,^38^ while the current study found inhibiting miR‐223‐3p by miR‐inhibitor cause a decrease expression of VEGFA, overexpression of miR‐223‐3p by miR‐mimic cause an increase expression of VEGFR2, this conflicted evidence indicated that miR‐223‐3p may act as bidirectional role in angiogenesis, this different function possibly depends on the cellular origin of tumour and nature of tumour cell transformation.^38^

## CONFLICT OF INTEREST

The authors confirm that there are no conflicts of interest.

## AUTHOR CONTRIBUTIONS

Limei Qin designed the experiments; Chunquan Ma and Qiang Fu performed the RNA sequencing and analysis; Zhili Li and Xiaoping Zhu performed the cellular experiments; Li Qin and Menglong Zhong performed the animal experiments; Nina Wang performed the interaction between F63 and miR‐223‐3p; Yanfeng Chen wrote the manuscript; and Dickson Adah and Xiaoping Chen reviewed the entire manuscript, with input from all authors.

## Data Availability

All data models generated or used during the study are available from the corresponding author by request.
